# Correction to “Kimura Disease With Eosinophilic Granulomatosis With Polyangiitis Successfully Treated by Mepolizumab”

**DOI:** 10.1002/ccr3.71409

**Published:** 2025-11-03

Du W. Dai R. Chen X. Tang W. “Kimura Disease With Eosinophilic Granulomatosis With Polyangiitis Successfully Treated by Mepolizumab,” *Clinical Case Reports* (2025) 13 no. 10: e71275, 10.1002/ccr3.71275.

In the beginning paragraph, the text “The authors received no specific funding for this work” was incorrect. This should be “This work was supported by NSFC No. 82170022.”

We apologize for this error.

